# Microscope-Integrated Indocyanine Green Videoangiography in Pediatric Spinal Dural Arteriovenous Fistula (SDAVF) Surgery: A Case Report on an Extremely Rare Vascular Malformation

**DOI:** 10.7759/cureus.86843

**Published:** 2025-06-27

**Authors:** Ehab Shabo, Ralf Clauberg, Motaz Hamed, Hartmut Vatter, Hannes Haberl, Sevgi Sarikaya-Seiwert

**Affiliations:** 1 Department of Neurosurgery, University Hospital Bonn, Bonn, DEU; 2 Department of Neuroradiology, University Hospital Bonn, Bonn, DEU; 3 Section of Pediatric Neurosurgery, Department of Neurosurgery, Schoen Clinic Vogtareuth, Vogtareuth, DEU; 4 Section of Pediatric Neurosurgery, Department of Neurosurgery, University Hospital Bonn, Bonn, DEU

**Keywords:** intraoperative indocyanine green, microscope, pediatric, spinal angiography, spinal dural arteriovenous fistulas

## Abstract

Spinal dural arteriovenous fistulas (SDAVFs) are exceptionally rare, acquired vascular malformations of the spinal cord, mostly affecting middle-aged to elderly men and typically presenting with progressive myelopathy. Given the diagnostic and therapeutic challenges of SDAVFs in children, we evaluated the feasibility and effectiveness of microscope-integrated indocyanine green (ICG) videoangiography as an intraoperative adjunct. While ICG use is well-documented in the surgical management of cranial aneurysms and adult spinal vascular malformations, its application in spinal arteriovenous malformations (AVM) in children remains limited and poorly characterized, and to date, its use in pediatric SDAVFs, especially, has not been reported. We present an exceptionally rare case of a pediatric SDAVF in a nine-year-old male patient who exhibited progressive, high-grade paraparesis and gait disturbances. The microsurgical disconnection of the fistula was performed at our institution. Intraoperative microscope-integrated ICG videoangiography was employed to localize the fistulous point at the Th7 level and to confirm the complete obliteration of the arteriovenous shunt in real time. No intraoperative or postoperative complications related to ICG administration were observed. Postoperative digital subtraction angiography (DSA) confirmed complete fistula closure. No recurrence or delayed complications were noted during a 70-month follow-up period. The results of this case report underscore the safety and effectiveness of intraoperative microscope-integrated ICG videoangiography for localizing and confirming the obliteration of pediatric SDAVF. This technique offers a promising alternative to routine postoperative DSA, reducing radiation exposure-associated risks in the pediatric population. Further studies on larger cohorts are warranted to validate these findings and standardize the use of ICG in pediatric spinal vascular surgery.

## Introduction

Spinal dural arteriovenous fistula (SDAVF) is a rare, acquired vascular disorder and represents the most common type of spinal vascular malformation [1,2]. The estimated incidence is 5-10 new cases per one million individuals annually [3]. SDAVFs predominantly affect elderly men, with a mean age at diagnosis between 55 and 60 years [4]. These lesions are typically solitary and most commonly located in the thoracolumbar region, spanning vertebral levels T6 to L2 [5].

The exact etiology of SDAVF remains unclear. The arteriovenous shunt is consistently in relation to the spinal nerve root, where arterial blood from the radiculomeningeal artery drains directly into a radicular vein at the dorsal surface of the dural root and is located within the dural layer of the spinal canal [4]. Spinal cord-supplying vessels are not primarily involved. The arterialization of the venous system leads to elevated spinal venous pressure and a reduction in the normal arteriovenous pressure gradient, resulting in impaired venous drainage. This venous congestion ultimately causes chronic spinal cord hypoxia, intramedullary edema, and progressive myelopathy [6-8].

The initial clinical presentation of SDAVF is often nonspecific, contributing to delays in diagnosis. Early symptoms commonly include sensory disturbances such as paresthesia, sensory loss, and gait instability. Patients may also experience radicular pain or nonspecific lower back pain without clear radicular distribution [9,10]. Late clinical symptoms include bowel and bladder incontinence with urinary retention, as well as erectile dysfunction. In advanced stages, SDAVF can lead to slowly progressive paraplegia [10,11].

Early diagnosis is crucial, as treatment outcomes are strongly influenced by the duration and severity of spinal cord involvement. The diagnosis of SDAVF is typically initiated with magnetic resonance imaging (MRI), with T2-weighted sequences revealing intramedullary hyperintensities indicative of congestive myelopathy. Dilated perimedullary vessels may appear as flow voids on T2-weighted or constructive interference in steady state (CISS) sequences [12,13]. Additionally, the non-invasive localization of the arteriovenous shunt using T1-weighted and gadolinium-enhanced magnetic resonance (MR) images is extremely helpful to guide the invasive conventional spinal digital subtraction angiography (DSA), which remains the diagnostic gold standard, and avoid unnecessary selective injections of all possible arterial feeders [14-16]. Therefore, spinal angiography currently plays a pivotal role in precisely identifying the fistula and localizing the site of the arteriovenous shunt, although this can be technically challenging in some cases [12].

Therapeutic options for SDAVF include either endovascular embolization using liquid embolic agents or the microsurgical disconnection of the fistula [17,18]. Surgical treatment has demonstrated high success rates, with fistula occlusion reported in up to 98% of cases, whereas the efficacy of endovascular approaches ranges from 25% to 75% depending on fistula anatomy and embolization technique [11,19]. Post-interventional spinal DSA remains the standard for confirming fistula obliteration in both treatment modalities [20].

Microscope-integrated near-infrared indocyanine green (ICG) videoangiography has been established as a valuable intraoperative tool in aneurysm surgery [21]. Furthermore, ICG videoangiography has been reported in adult spinal vascular surgery, including SDAVFs, in a limited number of cases to provide real-time intraoperative visualization of arteriovenous shunts, aiding in the precise identification and confirmation of successful fistula disconnection [22-24]. One small-cohort study even reported significantly improved surgical outcomes, reduced reoperation rates, and better clinical recovery compared to surgery without ICG or embolization alone [22]. However, there are no published reports of ICG application in pediatric patients.

Pediatric SDAVFs are extremely rare and often not considered in the initial differential diagnosis, which contributes to diagnostic delays [25]. Moreover, given the heightened sensitivity of pediatric patients to ionizing radiation [26] and the current reliance on postoperative spinal DSA as the gold standard for confirming complete fistula obliteration [27], there is a pressing need to explore and examine alternative intraoperative imaging modalities, such as ICG videoangiography, to minimize radiation exposure in this vulnerable population.

This case report aims to present the first documented pediatric SDAVF treated with intraoperative ICG videoangiography. We highlight its utility as a safe, rapid, and radiation-free method for the real-time confirmation of fistula disconnection. This is especially relevant given the as low as reasonably achievable (ALARA) principle guiding pediatric imaging practices.

ICG is a near-infrared fluorescent dye originally developed for assessing cardiocirculatory and hepatic functions, and it has also been employed in ocular angiography [28,29]. The dye exhibits absorption and emission peaks at approximately 805 nm and 835 nm, respectively, with an effective excitation wavelength range from 700 to 850 nm [29]. Following intravenous bolus injection, ICG rapidly binds to plasma globulins within 1-2 seconds, remaining confined to the intravascular space due to the high molecular weight of the protein complex [29]. When illuminated by a microscope-integrated near-infrared light source, ICG fluorescence enables the real-time visualization of vascular structures, including arteriovenous shunts. These properties make it suitable for intraoperative use without requiring complex equipment or training [21,29].

## Case presentation

Clinical presentation

A nine-year-old boy presented with a three-year history of progressive paraparesis, initially beginning at age 6, with the final diagnosis of SDAVF established at age 9. At the time of diagnosis, motor strength in the lower limbs was graded 2/5 on the Medical Research Council (MRC) scale, accompanied by marked spinal ataxia. Further neurological examination revealed a pathological Romberg sign with postural instability and a tendency to fall without lateralization. Lhermitte’s sign was absent. Sensory testing demonstrated impaired tactile perception in the lower extremities, while thermal sensation (warm and cold) was preserved. Sensory modalities in the upper extremities were intact. Coordination testing showed a normal finger-to-nose performance. However, the heel-to-shin test could not be reliably assessed bilaterally due to marked paraparesis of the lower limbs.

Diagnostic and surgical workflow

At the onset of symptoms, the differential diagnosis included a range of possibilities, notably inflammatory myelitis (e.g., transverse myelitis, neuromyelitis optica spectrum disorder {NMOSD}, and myelin oligodendrocyte glycoprotein antibody-associated disease {MOGAD}), hereditary myelopathies (such as hereditary spastic paraplegia or Friedreich’s ataxia), metabolic/nutritional disorders (e.g., vitamin B12 or copper deficiency), and compressive etiologies (including intramedullary tumors or congenital malformations). Cerebral pathologies such as cerebral paramedian tumors or vascular malformations were also considered.

According to documents at the time of the patient’s presentation at our institute, an early spinal MRI performed externally at symptom onset (at the age of six) was conducted without revealing pathological signs. The initial false-negative MRI results could be explained due to the absence of prominent venous arterialization at the early stages. A cranial MRI also conducted externally was unremarkable, and extensive laboratory investigations, including screening for metabolic, inflammatory, and hereditary etiologies, gradually helped to rule out other differential diagnoses.

Upon presentation to our institution, the child underwent a comprehensive holospinal MRI with all possible sequences, which demonstrated features consistent with SDAVF. To further delineate the fistula and confirm its anatomical configuration in a three-dimensional (3D) view, a spinal DSA was subsequently performed three days later under general anesthesia, in collaboration with a dedicated pediatric anesthesiologist and experienced neuroradiologist. The SDAVF at the T7 level was then definitively visualized and localized.

These combined imaging and diagnostic efforts allowed us to make a conclusive diagnosis and plan appropriate microsurgical intervention.

**Figure 1:**
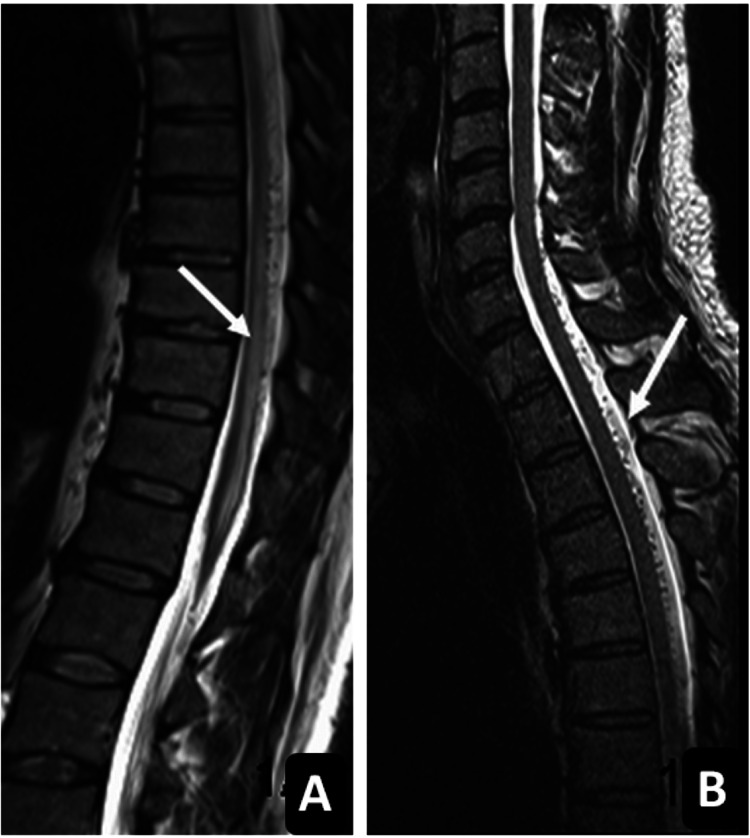
Sagittal T2-weighted MRI (A and B). In image A, a hyperintense intramedullary signal alteration from T7 to T12 represents edema (white arrow). In image B, the multiple flow voids in the dorsal subarachnoid space are distributed over almost the entire thoracic cord length, complying with pathological distended vessels (white arrow). MRI: magnetic resonance imaging

**Figure 2:**
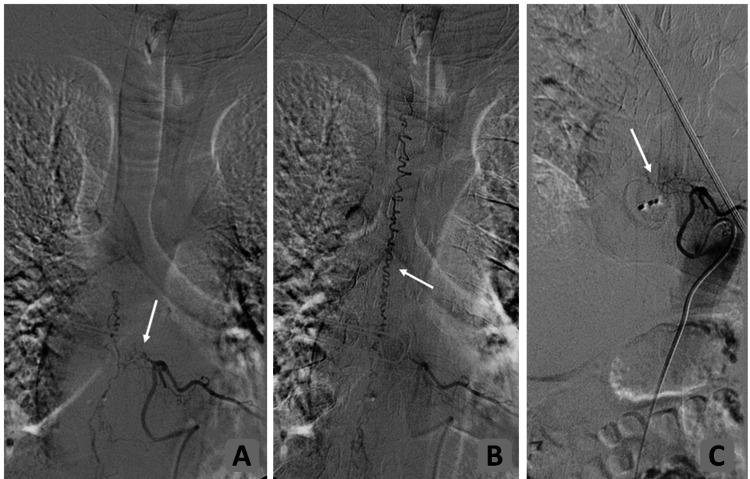
Preoperative (A and B) and postoperative (C) spinal DSA from the left side at the level T7. Image A shows the fistula located caudal of the T7 pedicle. The shunting zone is denoted by the white arrow. The main supply to the shunt is derived from the radiculomeningeal artery originating from the injected segmental artery. There is a marked alteration of the caliber of the vessels at the fistula site as the exact position of the shunt. In image B, contrast media highlights the dilated dorsal pial venous plexus, revealing cephalad-directed venous blood flow (white arrow). In image C, the arteriovenous shunt has been successfully occluded, indicating a complete cure. DSA: digital subtraction angiography

Postoperative spinal DSA was performed 10 days after surgery to validate intraoperative findings and confirm the complete obliteration of the fistula (Figure 1 and Figure 2).

**Figure 3:**
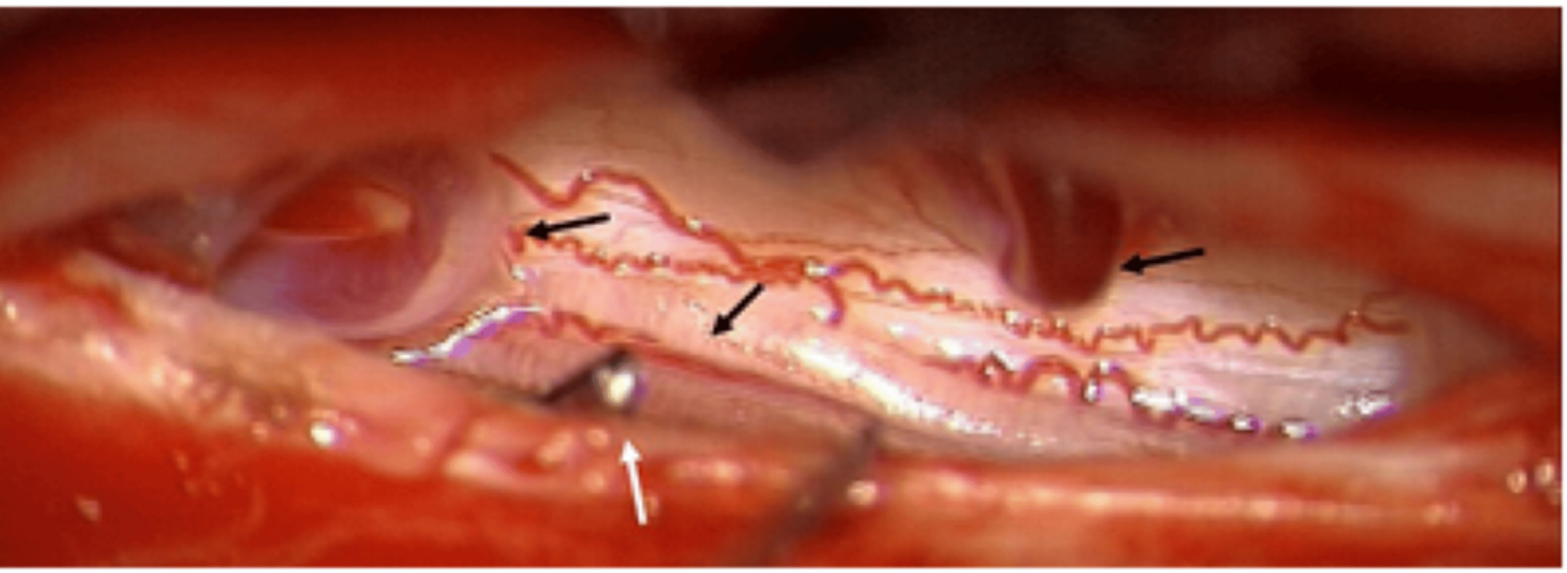
An intraoperative screenshot of the situs. The black arrows show the dilated dorsal pial venous plexus. The arteriovenous shunt is interrupted by an AVM clip (white arrow). AVM: arteriovenous malformation

Surgical intervention was performed under general anesthesia with the patient in a prone position. A level-specific hemilaminectomy (Th7) was carried out based on the preoperative localization. Following midline dural opening, the dorsal spinal cord surface and the fistulous connection were visualized under high magnification (Figure 3).

**Figure 4:**
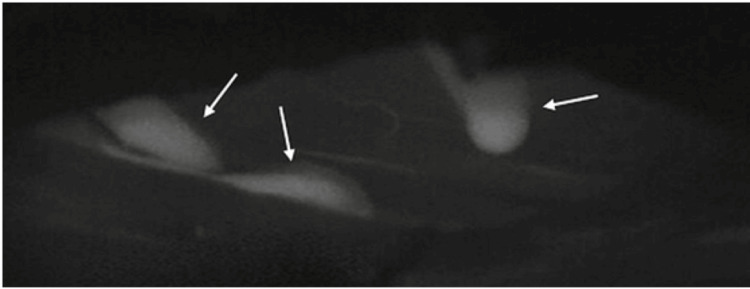
An intraoperative post-ICG injection screenshot of the intradural space after supplying intravenous ICG. The ICG videoangiography shows the dilated pial venous plexus and also the point of fistula (white arrows). ICG: indocyanine green

After the initial microsurgical inspection, a bolus of 25 mg ICG diluted in 5 mL saline, as pre-planned prior to surgery, was administered intravenously. Using a microscope equipped with near-infrared ICG visualization, the arteriovenous shunt and associated dilated perimedullary veins were visualized in real time. Following the microsurgical disconnection of the fistula and the coagulation of the draining vein, a second ICG injection was administered to confirm the complete obliteration of the shunt. The absence of ICG fluorescence in the dilated venous structures was considered the intraoperative confirmation of the successful and complete disconnection of the fistula (Figure 4).

Generally, ICG videoangiography has inherent constraints, such as limited tissue penetration depth, which may restrict visualization to superficial vascular structures. This limitation is particularly relevant in cases where the fistulous point or venous drainage is located deeper within the surgical field. Additionally, the short half-life of ICG may necessitate repeat injections during prolonged procedures or when multiple assessments are required. In our case, however, a single injection was sufficient to achieve a clear intraoperative visualization and confirmation of fistula disconnection.

Moreover, the administered ICG dosage was under close monitoring in a controlled intraoperative setting and determined in consultation with our pediatric anesthesiology team and falls well within accepted clinical practice parameters. No adverse reactions were observed during or after administration. We emphasize that patient safety was prioritized, and dosing decisions were tailored carefully to the child’s body weight and clinical condition.

Outcomes and follow-up

At the time of presentation, the patient exhibited severe paraparesis (MRC grade 2/5) with a symptom duration of approximately three years. Given the chronicity of spinal cord dysfunction, substantial neurological recovery was not anticipated. Instead, the primary objective of surgical intervention was to halt further clinical deterioration. Over a follow-up period of 70 months, the patient’s neurological status remained stable without any signs of further deterioration. Radiological follow-up also demonstrated sustained complete obliteration of the fistula with no evidence of recurrence or delayed complications. No additional surgical or endovascular procedures were required during the follow-up period.

Overall, microscope-integrated ICG videoangiography proved to be a reliable and effective tool for intraoperative navigation and real-time confirmation of fistula obliteration and therefore the assessment of surgical success. The absence of fluorescence in dilated venous structures after disconnection consistently correlated with complete fistula occlusion on postoperative spinal DSA.

While ICG videoangiography offers the advantage of real-time, radiation-free assessment during surgery, it remains inherently limited in depth penetration and field of view and thus may miss deeper or more complex vascular structures. In contrast, postoperative spinal DSA remains superior in terms of spatial resolution and ability to assess the entire vascular axis comprehensively. Therefore, although ICG provides high intraoperative specificity for confirming successful fistula disconnection when the vascular anatomy is accessible, it is currently still considered complementary to, rather than a replacement for, postoperative DSA in ensuring complete treatment verification.

## Discussion

Spinal dural arteriovenous fistulas (SDAVFs) in children are exceptionally rare and pose significant diagnostic and therapeutic challenges. These challenges stem from their nonspecific clinical presentation and slow progression and the anatomical complexity of spinal vascular structures. This often leads to misdiagnosis or delayed recognition, as in our case, where symptoms progressed over three years before a definitive diagnosis was made. Due to the rarity of this condition, literature on pediatric SDAVFs remains limited, and evidence-based management strategies are still evolving.

In adult population, DSA remains the gold standard for confirming the complete occlusion of the fistula following microsurgical intervention, offering high spatial resolution and comprehensive vascular assessment [20], and is considered relatively safe, with complication rates ranging from 1% to 3%, including rare instances of permanent neurological deficits [30,31]. However, translating this safety profile to pediatric populations requires caution. Children are more sensitive to ionizing radiation, require general anesthesia for DSA, and have smaller-caliber vessels that pose greater procedural challenges [32]. These considerations highlight the need for less invasive, safer, and equally beneficial imaging modalities in pediatric populations.

One such modality is MR angiography, which may help reduce the duration and radiation exposure of catheter angiography, particularly in patients with comorbidities such as renal insufficiency or advanced atherosclerosis [32]. Nevertheless, MR angiography, while non-invasive and less expensive, often lacks the spatial resolution and dynamic flow assessment of DSA, particularly in smaller-caliber pediatric vasculature [33].

The other modality is intraoperative ICG videoangiography, which is widely used in adult neurosurgery for the management of cerebral aneurysms and arteriovenous malformations (AVM) [21,28,29]. Intraoperative ICG videoangiography offers several potential advantages in the intraoperative setting. It provides real-time visualization of blood flow through exposed vessels using near-infrared fluorescence with a favorable safety and reliability profile, with severe side effects such as hypotension or arrhythmias occurring in only 0.05% of cases and moderate reactions such as pruritus or nausea in approximately 0.2% [34,35]. A further significant advantage of intraoperative ICG videoangiography lies in its ability to spare patients an additional postoperative DSA, which is particularly relevant in children as reducing ionizing radiation exposure, which could have sever risks in children compared to adult population, aligns with the ALARA principle (“as low as reasonably achievable”) in pediatric imaging [26,36,37]. Moreover, the immediate intraoperative feedback enhances surgical precision and confidence in the completeness of fistula disconnection, especially with the ability to apply a temporary clip, which allows a valuable assessment of the arteriovenous fistula prior to permanent occlusion, enabling the real-time confirmation of successful disconnection [37].

Nevertheless, ICG videoangiography has limitations. In contrast to postoperative DSA, it lacks the capacity to visualize deeper or multiple vascular feeders not directly exposed in the surgical field, offers relatively lower image resolution, does not provide three-dimensional visualization, may require repeated dye injections within short intervals potentially leading to false-positive findings, and relies predominantly on the surgeon’s subjective intraoperative judgment rather than the multidisciplinary evaluation typically involved in DSA interpretation [21,28,29,38,39].

However, despite its established role in adult vascular neurosurgical procedures, its application in pediatric spinal vascular lesions, especially SDAVFs, has not been reported, making our case a novel contribution.

In our patient, microscope-integrated ICG videoangiography allowed the direct intraoperative visualization of the arteriovenous shunt and its venous drainage. This enabled the immediate confirmation of successful disconnection following temporary clip application, which was later validated by postoperative DSA showing no residual or recurrent fistula. Notably, no adverse events or side effects related to ICG administration were observed.

While statistical power is lacking in this case report, a further limitation that must be acknowledged, the consistency of intraoperative ICG findings with postoperative DSA results and the absence of complications highlight the potential of this technique as a reliable, radiation-free adjunct in pediatric SDAVF surgery. Furthermore, the simplicity and rapid availability of this technique, requiring no extensive setup or specialized operator training, are additional advantages over postoperative spinal DSA, particularly in settings where radiation avoidance is a priority.

This case report adds to the growing discussion on optimizing imaging strategies for pediatric spinal vascular malformations. It highlights the potential of intraoperative ICG videoangiography as a safe, rapid, and effective intraoperative complementary tool to postoperative DSA, not only for intraoperative decision-making but also for potentially reducing the need for post-procedural angiography.

These findings seem promising. However, they stem from a single case and should be interpreted with caution. While larger prospective studies are necessary to validate these findings and determine whether ICG can fully replace postoperative DSA, the rarity of SDAVFs in children presents a significant challenge. Therefore, a multicenter, multinational prospective study would be the most appropriate approach to further evaluate the diagnostic accuracy, safety, and clinical impact of intraoperative ICG videoangiography in this unique patient population.

## Conclusions

The findings of this case suggest that microscope-integrated ICG videoangiography may serve as a safe, rapid, and effective intraoperative adjunct in the surgical management of pediatric SDAVFs. The technique enabled the real-time identification of the fistulous point and the intraoperative confirmation of disconnection without procedure-related complications. The consistency between intraoperative ICG and postoperative spinal DSA findings highlights the potential for this approach to complement or, in carefully selected cases, possibly reduce the need for routine postoperative spinal DSA, particularly in children where minimizing radiation exposure is a priority, though such conclusions should be drawn cautiously given the single-case nature of this report. Furthermore, the integration of ICG videoangiography into pediatric spinal vascular workflows appears feasible and may contribute to optimizing intraoperative decision-making. However, these observations are based on a single case and should be interpreted with caution. To establish the clinical utility, reliability, and generalizability of intraoperative ICG in pediatric SDAVF management, larger multicenter and prospective studies are warranted to validate these observations, assess cost-effectiveness, and further clarify the role of ICG in reducing imaging burden while maintaining surgical safety.

## References

[1] Kendall BE, Logue V (1977). Spinal epidural angiomatous malformations draining into intrathecal veins. Neuroradiology.

[2] Merland JJ, Riche MC, Chiras J (1980). Intraspinal extramedullary arteriovenous fistulae draining into the medullary veins. J Neuroradiol.

[3] Thron A (2001). [Spinal dural arteriovenous fistulas] (Article in German). Radiologe.

[4] Jellema K, Tijssen CC, van Gijn J (2006). Spinal dural arteriovenous fistulas: a congestive myelopathy that initially mimics a peripheral nerve disorder. Brain.

[5] Schaat TJ, Salzman KL, Stevens EA (2002). Sacral origin of a spinal dural arteriovenous fistula: case report and review. Spine (Phila Pa 1976).

[6] Kataoka H, Miyamoto S, Nagata I, Ueba T, Hashimoto N (2001). Venous congestion is a major cause of neurological deterioration in spinal arteriovenous malformations. Neurosurgery.

[7] Hurst RW, Kenyon LC, Lavi E, Raps EC, Marcotte P (1995). Spinal dural arteriovenous fistula: the pathology of venous hypertensive myelopathy. Neurology.

[8] Hassler W, Thron A (1994). Flow velocity and pressure measurements in spinal dural arteriovenous fistulas. Neurosurg Rev.

[9] Koenig E, Thron A, Schrader V, Dichgans J (1989). Spinal arteriovenous malformations and fistulae: clinical, neuroradiological and neurophysiological findings. J Neurol.

[10] Jellema K, Canta LR, Tijssen CC, van Rooij WJ, Koudstaal PJ, van Gijn J (2003). Spinal dural arteriovenous fistulas: clinical features in 80 patients. J Neurol Neurosurg Psychiatry.

[11] Van Dijk JM, TerBrugge KG, Willinsky RA, Farb RI, Wallace MC (2002). Multidisciplinary management of spinal dural arteriovenous fistulas: clinical presentation and long-term follow-up in 49 patients. Stroke.

[12] Krings T, Lasjaunias PL, Hans FJ (2007). Imaging in spinal vascular disease. Neuroimaging Clin N Am.

[13] Gilbertson JR, Miller GM, Goldman MS, Marsh WR (1995). Spinal dural arteriovenous fistulas: MR and myelographic findings. AJNR Am J Neuroradiol.

[14] Bowen BC, Fraser K, Kochan JP, Pattany PM, Green BA, Quencer RM (1995). Spinal dural arteriovenous fistulas: evaluation with MR angiography. AJNR Am J Neuroradiol.

[15] Farb RI, Kim JK, Willinsky RA (2002). Spinal dural arteriovenous fistula localization with a technique of first-pass gadolinium-enhanced MR angiography: initial experience. Radiology.

[16] Mull M, Nijenhuis RJ, Backes WH, Krings T, Wilmink JT, Thron A (2007). Value and limitations of contrast-enhanced MR angiography in spinal arteriovenous malformations and dural arteriovenous fistulas. AJNR Am J Neuroradiol.

[17] Huffmann BC, Gilsbach JM, Thron A (1995). Spinal dural arteriovenous fistulas: a plea for neurosurgical treatment. Acta Neurochir (Wien).

[18] Krings T, Mull M, Gilsbach JM, Thron A (2005). Spinal vascular malformations. Eur Radiol.

[19] Niimi Y, Berenstein A, Setton A, Neophytides A (1997). Embolization of spinal dural arteriovenous fistulae: results and follow-up. Neurosurgery.

[20] Munshi I, Macdonald RL, Weir BK (1999). Intraoperative angiography of brain arteriovenous malformations. Neurosurgery.

[21] Raabe A, Nakaji P, Beck J (2005). Prospective evaluation of surgical microscope-integrated intraoperative near-infrared indocyanine green videoangiography during aneurysm surgery. J Neurosurg.

[22] Koyalmantham V, Kale SS, Devarajan LJ (2020). Patient outcomes following obliteration of spinal dural arteriovenous fistula and the role of indocyanine green angiography videoangiography (ICG-VA) during surgery. Neurol India.

[23] Oh JK, Shin HC, Kim TY (2011). Intraoperative indocyanine green video-angiography: spinal dural arteriovenous fistula. Spine (Phila Pa 1976).

[24] Murakami T, Koyanagi I, Kaneko T, Iihoshi S, Houkin K (2011). Intraoperative indocyanine green videoangiography for spinal vascular lesions: case report. Neurosurgery.

[25] Krings T, Geibprasert S (2009). Spinal dural arteriovenous fistulas. AJNR Am J Neuroradiol.

[26] Granata C, Sofia C, Francavilla M (2025). Let's talk about radiation dose and radiation protection in children. Pediatr Radiol.

[27] Raymaekers V, Rodríguez-Hernández A, Pegge SA, Menovsky T, Meijer FJ, Boogaarts JH (2025). Diagnostic accuracy of 4D-MRA for the detection and localization of spinal dural arteriovenous fistulas: a systematic review and meta-analysis. World Neurosurg.

[28] Balamurugan S, Agrawal A, Kato Y, Sano H (2011). Intra operative indocyanine green video-angiography in cerebrovascular surgery: an overview with review of literature. Asian J Neurosurg.

[29] Raabe A, Beck J, Gerlach R, Zimmermann M, Seifert V (2003). Near-infrared indocyanine green video angiography: a new method for intraoperative assessment of vascular flow. Neurosurgery.

[30] Derdeyn CP, Moran CJ, Cross DT, Grubb RL Jr, Dacey RG Jr (1995). Intraoperative digital subtraction angiography: a review of 112 consecutive examinations. AJNR Am J Neuroradiol.

[31] Ali S, Cashen TA, Carroll TJ, McComb E, Muzaffar M, Shaibani A, Walker MT (2007). Time-resolved spinal MR angiography: initial clinical experience in the evaluation of spinal arteriovenous shunts. AJNR Am J Neuroradiol.

[32] Luetmer PH, Lane JI, Gilbertson JR, Bernstein MA, Huston J II, Atkinson JL (2005). Preangiographic evaluation of spinal dural arteriovenous fistulas with elliptic centric contrast-enhanced MR angiography and effect on radiation dose and volume of iodinated contrast material. AJNR Am J Neuroradiol.

[33] Song P, Qin J, Lun H, Qiao P, Xie A, Li G (2017). Magnetic resonance imaging (MRI) and digital subtraction angiography investigation of childhood moyamoya disease. J Child Neurol.

[34] Cochran ST, Bomyea K, Sayre JW (2001). Trends in adverse events after IV administration of contrast media. AJR Am J Roentgenol.

[35] Hope-Ross M, Yannuzzi LA, Gragoudas ES (1994). Adverse reactions due to indocyanine green. Ophthalmology.

[36] Borders HL, Barnes CL, Parks DC, Jacobsen JR, Zhou Y, Hasselquist BE, Betz BW (2012). Use of a dedicated pediatric CT imaging service associated with decreased patient radiation dose. J Am Coll Radiol.

[37] Karahalios K, Scherschinski L, Srinivasan VM (2025). Intraoperative indocyanine green videoangiography versus postoperative catheter angiography to confirm microsurgical occlusion of cranial dural arteriovenous fistulas. Clin Neurol Neurosurg.

[38] de Oliveira JG, Beck J, Seifert V, Teixeira MJ, Raabe A (2007). Assessment of flow in perforating arteries during intracranial aneurysm surgery using intraoperative near-infrared indocyanine green videoangiography. Neurosurgery.

[39] Dashti R, Laakso A, Niemelä M, Porras M, Hernesniemi J (2011). Microscope integrated indocyanine green video-angiography in cerebrovascular surgery. Acta Neurochir Suppl.

